# Where the Road Ends, Yaws Begins? The Cost-effectiveness of Eradication versus More Roads

**DOI:** 10.1371/journal.pntd.0003165

**Published:** 2014-09-25

**Authors:** Christopher Fitzpatrick, Kingsley Asiedu, Jean Jannin

**Affiliations:** Department of Control of Neglected Tropical Diseases, World Health Organization, Geneva, Switzerland; University of Washington, United States of America

## Abstract

**Introduction:**

A disabling and disfiguring disease that “begins where the road ends”, yaws is targeted by WHO for eradication by the year 2020. The global campaign is not yet financed. To evaluate yaws eradication within the context of the post-2015 development agenda, we perform a somewhat allegorical cost-effectiveness analysis of eradication, comparing it to a counterfactual in which we simply wait for more roads (the end of poverty).

**Methods:**

We use evidence from four yaws eradication pilot sites and other mass treatment campaigns to set benchmarks for the cost of eradication in 12 known endemic countries. We construct a compartmental model of long-term health effects to 2050. Conservatively, we attribute zero cost to the counterfactual and allow for gradual exit of the susceptible (at risk) population by road (poverty reduction). We report mean, 5^th^ and 95^th^ centile estimates to reflect uncertainty about costs and effects.

**Results:**

Our benchmark for the economic cost of yaws eradication is uncertain but not high –US$ 362 (75–1073) million in 12 countries. Eradication would cost US$ 26 (4.2–78) for each year of life lived without disability or disfigurement due to yaws, or US$ 324 (47–936) per disability-adjusted life year (DALY). Excluding drugs, existing staff and assets, the financial cost benchmark is US$ 213 (74–522) million. The real cost of waiting for more roads (poverty reduction) would be 13 (7.3–20) million years of life affected by early-stage yaws and 2.3 (1.1–4.2) million years of life affected by late-stage yaws.

**Discussion:**

Endemic countries need financing to begin implementing and adapting global strategy to local conditions. Donations of drugs and diagnostics could reduce cost to the public sector and catalyze financing. Resources may be harnessed from the extractive industries. Yaws eradication should be seen as complementary to universal health coverage and shared prosperity on the post-2015 development agenda.

## Author Summary

A disabling and disfiguring disease that “begins where the road ends” (among poor and isolated communities), yaws is targeted by WHO for eradication by the year 2020. The global campaign is not yet financed. We provide benchmarks for the cost and health effects of global yaws eradication, based on evidence from four yaws eradication pilot sites and other mass treatment campaigns. We suggest that a global yaws eradication campaign could be established with a relatively modest investment in the period 2015–2020 — as little as US$ 100 million in the 12 known endemic countries. Eradication would cost about US$ 26 for each additional year of life lived without disability or disfigurement due to yaws between the years 2015 and 2050. The real cost of not doing anything but wait for more roads (the end of poverty) would be about 15 million years of life needlessly affected by disability and disfigurement. We expect that yaws eradication will be cost-effective. Importantly, from the perspective of universal health coverage, it will benefit some of the world's least well off citizens. Yaws eradication should therefore be seen as complementary to universal health coverage and shared prosperity on the post-2015 development agenda.

## Introduction

Yaws is one of two neglected tropical diseases (NTDs) targeted by the World Health Organization (WHO) for eradication. A 2013 World Health Assembly resolution calls for its eradication by the year 2020. A disabling and disfiguring disease that “begins where the road ends” it is found primarily among poor and isolated communities in warm, humid and tropical forest areas of Africa, South-East Asia and the Western Pacific. It is caused by a bacterium (*Treponema pallidum ssp pertenue*) related to syphilis but is not sexually-transmitted and mostly afflicts children. In its primary and secondary (early) stages it causes unsightly and often painful lesions of the skin (especially face and feet), cartilage and bones. About 10% of untreated cases suffer tertiary (late-stage) yaws, with permanent disability and disfigurement of the face, lower limbs and hands. In 1950, WHO estimated that 160 million people were infected with yaws [1]. Between 2008 and 2012 more than 300 000 new cases were reported to WHO [2]. Reporting yaws is not mandatory, however, and so the full burden of the disease is not currently known.

In the field, diagnosis is primarily based on epidemiology and clinical symptoms. Laboratory-based serological tests are widely used to confirm clinical cases. The same tests are used to confirm syphilis but cannot distinguish between the two diseases. Recently, a rapid syphilis test has been demonstrated to be effective in confirming yaws and can be used in the field [3]. There is no vaccine for yaws. Prevention is based on the interruption of transmission through early diagnosis and treatment of individual cases and total (mass) or targeted treatment of affected populations. The epidemiology of the disease, the history of its control and the feasibility of eradication are described in detail elsewhere [1] [4] [5] [6] [7] [8] [9] [10]. WHO's strategy for yaws eradication is based on single-dose oral treatment with azithromycin at 30 mg/kg (maximum 2 g) [11]. Because of the simplicity and convenience, it is now preferred to the traditional treatment with injectable benzathine penicillin.

Transmission has been shown to be interrupted with one or two rounds of treatment at high levels of population coverage [1]. The approach is known as total community treatment (TCT) – treatment of an entire endemic community irrespective of the number of active clinical cases. Total targeted treatment (TTT) – treatment of all active clinical cases and their contacts – is carried out to “mop-up” any cases that were missed in the TCT round. The definition of contacts may vary between settings, but normally includes household members and, in the case of school-age children, classmates. Confirmation of clinical cases during TCT (for follow-up TTT) may be carried out using a rapid dual point-of-care treponemal and non-treponemal serological test. “Proof of concept” pilot projects in Congo (Bétou and Enyellé districts), Papua New Guinea (Lihir island), Vanuatu (Tafea Province) and Ghana (West Akyem district) were successfully concluded in 2012 and 2013.

The tools exist for WHO and its partners to follow the global Guinea worm disease eradication campaign in eradicating another NTD. And yet, the effort is not financed, with no cash or in-kind donations yet received for a global yaws eradication campaign. As with other diseases of poverty, there is a tendency to hope that poverty reduction will resolve the problem. Unfortunately, the history of yaws suggests that a more concerted effort will be required. The dismantling of vertical yaws programs after 1964 led to a resurgence of yaws in the late 1970s, even as poverty rates declined [1]. To evaluate whether yaws eradication is a good investment within the broader post-2015 development agenda, we perform a cost and somewhat allegorical cost-effectiveness analysis of eradication, comparing it to a counterfactual in which we simply wait for more roads (the end of poverty).

## Methods

In this section we describe the model and parameters for the cost-effectiveness analysis. We use evidence from four yaws eradication pilot sites and other mass treatment campaigns to set benchmarks for the cost of an eradication campaign in 12 known endemic countries in 2015–2020. Conservatively, we assign zero cost to the counterfactual of waiting for more roads (the end of poverty). We develop a compartmental (Markov) model of primary, secondary and tertiary stage infection for the period 2015–2050. We incorporate gradual exit of the susceptible (at risk) population by road (poverty reduction). Given considerable uncertainty about most of the model parameters, we perform probabilistic sensitivity analysis (Monte Carlo simulation) using RStudio version 0.98.507 for R version 3.0.2. [12] [13].

The discount rate on costs is 3–6% per year (uniform distribution); the discount rate on health effects is 0–3% per year [14]. Best, low and high estimates correspond to the mean, 5^th^ and 95^th^ centile values from 1000 simulations.

### Population at risk

A review of the literature from 1950 to 2013 indicates that at least 85 countries have reported yaws [1]. Ecuador and India reported interruption of transmission of the disease in 2003 and 2006 respectively. 12 countries currently reporting cases to WHO require technical assistance and financing: Benin, Cameroon, Central African Republic, Congo, Cote d'Ivoire, Democratic Republic of the Congo (DRC), Ghana, Indonesia, Papua New Guinea, Solomon Islands, Togo and Vanuatu. In 71 countries where no recent data are available, the absence of the disease needs to be verified.

Expert opinion puts the population at risk for yaws at a minimum of 5 percent of the populations of ten of the twelve known endemic countries [15]. The exceptions are the small island states of Solomon Islands and Vanuatu, where 100 percent of the population is assumed at risk [16]. For an upper bound on population at risk we employ data from the G-Econ 4.0 (May 2011) database [17]. G-Econ provides demographic and geophysical data for one-degree longitude by one-degree latitude cells – approximately 100 km by 100 km or the same size as most second administrative level boundaries. We summed the populations living in cells satisfying the following conditions favorable to the transmission of yaws: 1) average precipitation (mm per year) >500; 2) average annual temperature (degrees Celsius) >20; 3) tropical forest or woodland; and 4) population density per km^2^ <100 [18]. We adjusted the reported 2005 populations to 2015 using rural population projections [16].

In addition to demographic and geophysical data, G-Econ contains estimates of Gross Domestic Product (GDP) at the level of cells. We use Gross Cell Product (GCP) in the results section of this paper to assess the economic productivity of the lands on which populations at risk for yaws live and establish a threshold by which to assess cost-effectiveness.

### Cost of drugs and diagnostics

To kick-start the pilot project, WHO procured limited quantities of generic azithromycin (Medopharm, India) at US$ 0.17 per 500 mg tablet. We used population data disaggregated by age to estimate dosage: 0–4 years (500 mg), 5–9 years (1000 mg), 10–14 years (1500 mg) and 15 years and older (2000 mg) [16]. Based on experience from the pilot sites, TTT may be required. The number of index cases requiring mop-up is determined by the coverage, eligibility and cure rates in the model of health effects (described below). For the purposes of the cost benchmarks, we assumed based on experience in India that mop-up reaches the active index case plus 10–20 close contacts that include the secondary cases. For the purposes of detailed planning and budgeting, local evidence will be needed. We assume that a 10% buffer stock is required.

For the pilot, WHO also procured rapid dual non-treponemal and treponemal point-of-care serological tests (Chembio Diagnostic System Inc., New York, USA) at a negotiated price of US$ 2 per test. The number of clinical cases that may be serologically tested during TCT and TTT is determined by the model of health effects (below), with an allowance for clinical misdiagnosis of yaws-like lesions that increases the total number of tests by 10–30%. We have not estimated the cost of more expensive molecular tests (polymerase chain reaction) to monitor for drug resistance or to confirm eradication. We assume that surveillance (below) is clinical surveillance and we do not include costs for any additional tests.

### Cost of delivery

Experience from the pilot sites suggests that the cost of delivery will vary considerably across endemic countries. In some pilot sites, the cost per person was relatively low. On the island of Lihir (Papua New Guinea), the financial cost of TCT of 16 941 people was about US$ 25 800 or US$ 1.52 per person. (personal communication with Oriol Mitja) This first round was completed in 40 days. Human resources included community and public health workers, a program coordinator, a nurse, a laboratory technician (half-time) and a driver. Six months later, the financial cost of TTT was US$ 11 400 or US$ 0.71 per person. Mop-up was completed in only 14 days because there was no serological testing and because fewer drugs were administered. In Ghana, the cost of TCT was even lower, but the program depended heavily on the contribution of volunteers.(personal communication with Abdul Aziz Abdulai)

In other sites, the cost per person treated was relatively high. In Tafea province (Vanuatu), the financial cost of TCT of 41 509 people distributed over five remote islands with very weak health and road infrastructure was about US$ 265 300 or US$ 8.27 per person. (personal communication with Jacob Kool) This amount included upfront investment in communication-for-behavioral-impact (COMBI). COMBI is thought to have improved acceptance rates and general hygiene and thereby reduced the need for a mop-up round. Likewise in the remote Bétou and Enyellé districts of the Congo, upfront equipment costs pushed unit costs into the high single digits. The team offered a mobile clinic and measles vaccination (requiring a costly cold-chain). (personal communication with Matthew Coldiron).

We did not assume that unit costs from the pilot sites were generalizable to other settings. In order to better understand the drivers of costs across settings, we reviewed the rather more expansive literature on the cost of mass drug administration (MDA) to control and eliminate other NTDs: lymphatic filariasis (LF), schistosomiasis, soil-transmitted helminthiasis (STH), onchocerciasis and trachoma. The 25 identified studies are referenced in the Supporting Information (Table S1). We extracted both financial (F) and economic (E) costs: planning, mapping and training activities (F&E), drug shipment (F&E), vehicles that were rented (F&E) or borrowed from other programs (E), fuel and vehicle maintenance (F&E), per diems (F&E), project staff salaries (F&E), Ministry of Health staff time (E), office space (E), utilities (F&E) and supplies (F&E). We removed drugs from the cost of delivery. Capital cost annualization and overhead cost allocation were retained from the individual studies. We did not consider the cost of volunteer time because most studies did not report it. We extracted also the number of people treated and GDP per capita.

We converted unit costs to constant 2012 US$ and ran multivariate regressions on the number of people treated to capture economies of scale, on GDP per capita (constant 2012 US$) to capture differences in the quality and complexity of inputs, and on population density to capture differences in logistical difficulty. We opted for study/site fixed effects and a log-log specification. Regression results based on 103 observations from 57 study-countries are available in Supporting Information (Table S2). These results were used to generate country-specific benchmarks for the economic unit cost of TCT. With the mean and standard error of the log prediction, we simulated and re-transformed 1000 values and extracted the mean, 5^th^ and 95^th^ centile values for the best estimate and 90% credible interval (CI).

We used the same approach to generate benchmarks for the financial unit cost, using studies with financial cost estimates. In our review, financial costs were on average 66% (interquartile range 28–86%) of economic costs. Both economic and financial unit cost benchmarks are available in the Supporting Information (Table S3). All costs in this paper refer to economic costs, unless otherwise specified.

We assumed, based on experience from Lihir that the cost of the TTT mop-up (if required) would be 30–50% of the cost of TCT. This assumption will be revisited as evidence comes in from other sites.

### Cost of surveillance

We reviewed the literature from similar programs to estimate the cost of surveillance following TCT and TTT. The (economic) cost of prevalence surveys for trachoma was about US$ 1600–28 000 per district of 100 000–250 000 inhabitants in 2013 prices [19]. Only 6% of this cost was for supplies. In probabilistic sensitivity analysis, we assumed surveillance costs of US$ 2000–30 000 per 100 000 population at risk. We assumed clinical surveillance and did not include costs for any additional tests (see cost of diagnostics, above). Surveillance is one of the aspects of yaws eradication that may require the most adaptation to local conditions. Yaws elimination in India and Guinea worm disease elimination in most countries used rumor investigation, including cash rewards of between US$ 10–1000 for the reporting of (subsequently confirmed) cases. In India, the cost of rewards was small relative to that of serological surveys.

### Health effects

**Figure 1:**
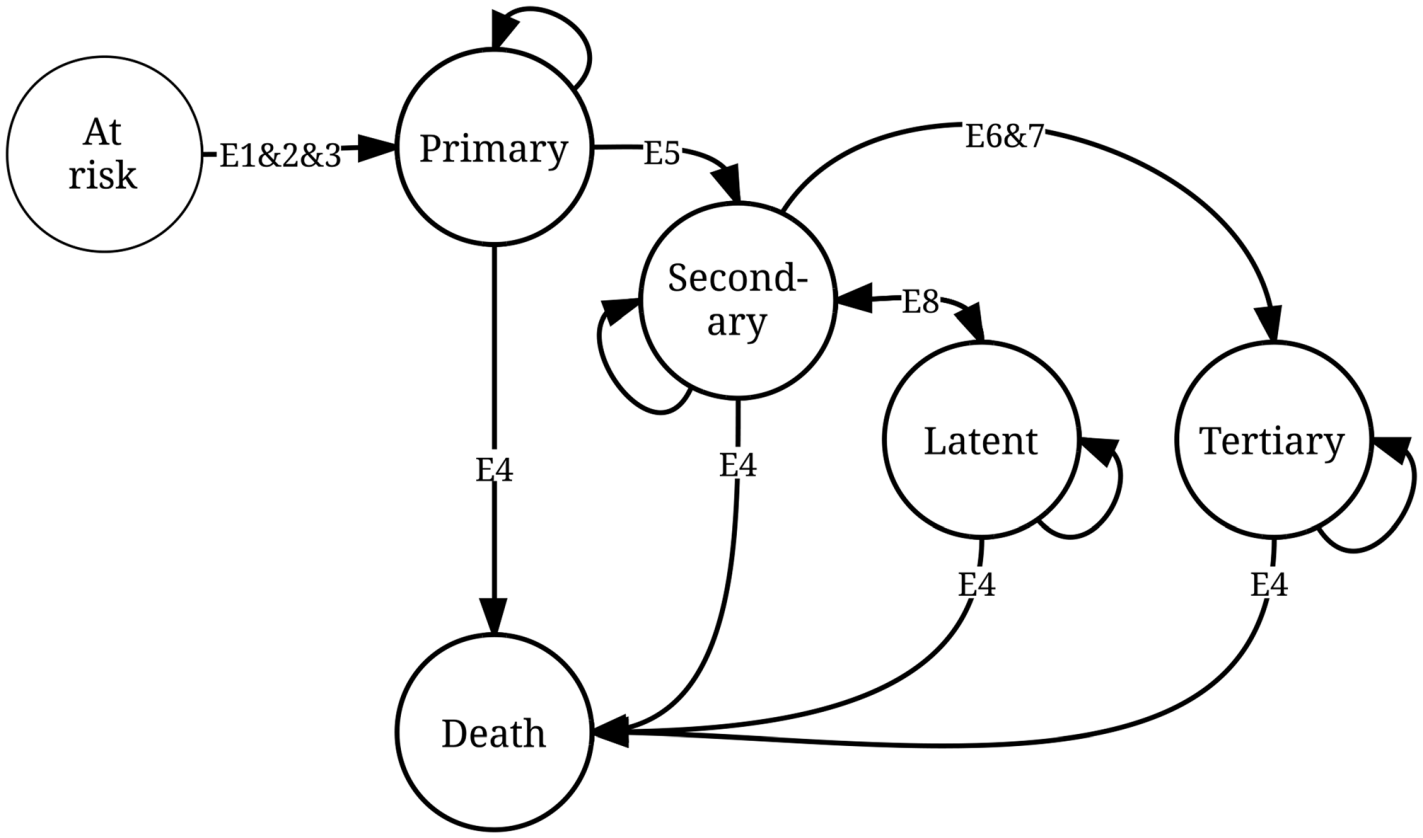
Compartmental (Markov) model of primary, secondary/latent and tertiary yaws. See Table 1 for sources and comments related to the epidemiological parameters E1–E8. The eradication scenario and counterfactual are differentiated by the programmatic parameters P1–P3, also in Table 1, which allow for cure and return by primary and secondary/latent cases to the susceptible (at risk) population.

Our compartmental (Markov) model is depicted in Figure 1. The population at risk moves to or through one of five possible states: primary, secondary, latent and tertiary yaws, or death (the terminal state). Ours is not the first compartmental model of yaws transmission [20]. But it is the first to distinguish between the stages of infection, and the first used in a cost-effectiveness analysis.

Start values for the model are based on the maximum number of new cases reported in any given year 2008–2012 [21]. We assumed conservatively that reported cases represented 30–90% of true incident cases. Based on experience with Buruli ulcer, another NTD affecting skin in poor and isolated populations, the number may be as low as 7% in the Democratic Republic of Congo and 18% in Cameroon [22] [23]. Transition probabilities from one state to another are determined by epidemiological parameters, with distributions for probabilistic sensitivity analysis (Table 1). We converted rates and durations into probabilities. We converted all probabilities in half-year (6-month) cycle probabilities.

**Table 1:** Epidemiological and programmatic parameters for the compartmental model.*

		Min	Max	Comment
**Epidemiological parameters for both the eradication campaign and counterfactual**				
**E1**	Basic reproduction number (R_0_)	0.9	0.999	This is the number of secondary cases generated by a single index case in the susceptible population, over the period given by **E2**. This is a conservative assumption for cost-effectiveness of the eradication campaign; the resurgence of yaws in the late 1970s would suggest an R_0_>1.
**E2**	Generation time (years)	0.08	5	The incubation period is less than 1 month on average and infectious relapses can happen for up to 5 years [10].
**E3**	Annual exit rate of the susceptible (at risk) population (annual reduction in R_0_)	2%	7%	50th and 75th centile values for the average rate of decline in the poverty headcount of 98 developing countries in the 15-year period 1999–2013 [16] [24]. This is an optimistic scenario for the annual decrease in the population at risk (or R_0_) that would occur even in the absence of an eradication campaign.
**E4**	Adult mortality of the susceptible and infected population, as a proportion of the adult mortality rate of the general population (probability of dying between 15 to 60 years)	1	1.2	Yaws does not affect mortality, but mortality may be higher in poor, rural communities than in the general population. Adult mortality rate of the general population is a country-specific value [21].
**E5**	Duration of primary stage before onset of secondary/latent stage (years), without treatment	0.25	0.5	Primary lesions usually heal after 3–6 months; secondary lesions appear a few weeks after the primary lesion and only 9–15% of patients have a primary lesion that persists at the onset of the secondary stage [10].
**E6**	Probability of progression from secondary to tertiary stage, without treatment	6%	10%	This is an arbitrary but conservative range around the best estimate (10%) reported in the literature [10]. The probability is for the period given by **E7**.
**E7**	Duration of primary and secondary yaws before onset of tertiary yaws (years), without treatment	5	10	E5 and E7 are then used to determine the duration of secondary/latent yaws [10].
**E8**	Ratio of latent to non-latent among secondary yaws cases	2	6	This is used to derive the percentage of secondary yaws cases that are latent (and conservatively assumed to be asymptomatic) [9] [10].
**Programmatic parameters for the eradication campaign**				
**P1**	Coverage (TCT round)	90%	99%	This is based on experience in the pilot sites [15]. Coverage in TTT rounds is assumed to be 100% of index cases and their close contacts.
**P2**	Eligibility for treatment (TCT round)	98%	99%	This is based on age-disaggregated population data [16]. Infants under 6 months of age are ineligible for treatment with azithromycin. Eligibility in TTT rounds is assumed to be 100%.
**P3**	Cure rate	Normal distribution; see Comment.		Mean = 85.5% and standard deviation = 0.031. Based on primary endpoint (cure at 6 months) of the intention-to-treat population from a randomized controlled trial in Papua New Guinea [30].

*See Figure 1 for a graphical representation of the model.

For both the eradication scenario and counterfactual, we made an optimistic assumption that the susceptible (at risk) population and, by extension, the basic reproduction number will decrease 2–7% per year, as a result of more roads (poverty reduction). These are the 50^th^ and 75^th^ centile values for the average annual rate of decline in the dollar-a-day poverty headcount of 98 developing countries over 1999–2013 [16] [24]. These correspond roughly to the values for India and China, respectively. This is an optimistic assumption resulting in a conservative estimate of cost-effectiveness.

The eradication scenario has additional, programmatic assumptions related to coverage, eligibility (for treatment) and cure rates (Table 1). Covered, eligible and cured individuals in the primary and secondary states return to the susceptible population. Tertiary yaws is irreversible. The model assumes that TTT will treat all index cases and their contacts — this is a simplification but has no major effect on the model. The model has not been constructed to prove the feasibility of eradication, as this has already been done in the field. Future refinements of the model could, however, help identify the conditions under which it is more cost-effective to follow TCT with another round of TCT rather than TTT.

We allowed the model to burn in over a period of 10 years, the maximum duration of progression to tertiary disease. The model was run to the year 2050 to capture some of the longer-term benefits of eradication. In reality the benefits of eradication could extend well beyond 2050. We summed the number of (discounted) life-years spent in the primary and secondary (early-stage) and tertiary (late-stage) states, and compared the eradication scenario results to those of the counterfactual.

There are no specific disability weights for early or late-stage yaws. We calculated disability-adjusted life-years (DALYs) using weights for comparable conditions [25]. We used 0.029 (0.016–0.048) for early stage yaws based on disfigurement level 1 with itch or pain, described as: “a slight, visible physical deformity that is sometimes sore or itchy. Others notice the deformity, which causes some worry and discomfort.” This range contains an earlier point estimate of 0.048 for secondary syphilis [26]. We used 0.398 (95% CI 0.271–0.543) for late-stage yaws based on disfigurement level 3, described as: “an obvious physical deformity that makes others uncomfortable, which causes the person to avoid social contact, feel worried, sleep poorly, and think about suicide.” This range contains an earlier point estimate of 0.283 for tertiary syphilis.

Our analysis does not take into account the potential knock-on benefits of total community treatment with azithromycin for trachoma, chancroid, chlamydia, syphilis, gastrointestinal and respiratory tract infections or malaria, nor of any of the other health services delivered during the campaigns. Reductions in child-mortality have been associated with mass administration of azithromycin for trachoma, but awaits confirmation by a randomized controlled trial [27]. In Vanuatu, the pilot sites saw a dramatic decrease in the number of diarrheal cases and all-cause hospitalizations.(personal communication, Jacob Kool).

## Results

### Population at risk

**Figure 2:**
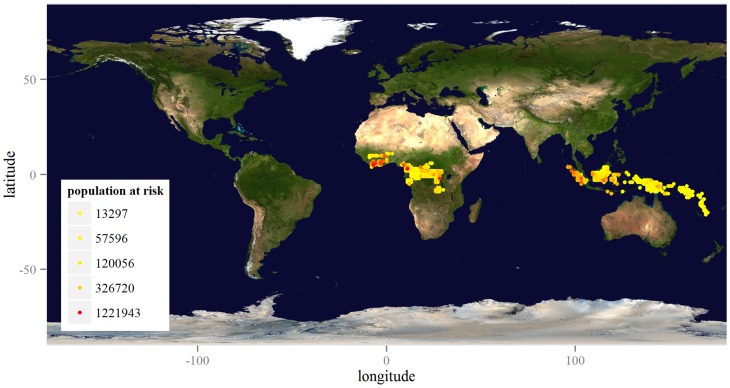
Geographic distribution of the estimated population at risk in 12 known endemic countries. The legend gives the quintile values for the population at risk living within one-degree latitude by one-degree longitude cells (approximately 100 km by 100 km). Map credit: NASA Goddard Space Flight Center Image by Reto Stöckli (land surface, shallow water, clouds). Available at http://visibleearth.nasa.gov/view.php?id=57752.

Expert opinion puts the minimum population at risk for yaws in the 12 known endemic countries in 2015 at 21 million. Using G-Econ data, we calculate that as many as 74 million people live under conditions favorable to yaws infection, as mapped by Figure 2. The upper bound on the range of estimates produced for Indonesia comes close to that obtained in a recent, more detailed exercise by the national program.

Extending this analysis to the 71 countries where cases are known to have occurred historically, we estimate that the population in need of verification of the absence of the disease is 210 million. In what follows, we calculate only the cost of surveillance for this population.

### Costs

Including buffer stock and mop-up, 75 (60–92) million grams of azithromycin are estimated to be required during 2015–2020. At US$ 0.17 per 500 mg tablet, the cost would be US$ 28 (22–34) million. The number of serology tests required for confirmation of clinical cases in the 12 endemic countries is estimated at 0.4 (0.2–0.5) million, at a cost of US$ 0.7 (0.4–1.1) million.

Best estimates from the regression models of the economic unit cost of delivery suggest a range, depending on the country, of US$ 0.20–10.41 per person. See Supporting Information for country-specific economic and financial unit cost benchmarks. The economic cost benchmarks imply that delivery would cost US$ 314 (31–1009) million. The financial cost would be lower. Both economic and financial cost benchmarks are reported in Table 2.

**Table 2:** Cost benchmarks for the 12 countries of known endemicity.

	Economic			Financial		
	Best	Low	High	Best	Low	High
Drugs	28	22	34	28	22	34
Diagnostics	0.7	0.4	1.1	0.7	0.4	1.1
Delivery	314	31	1008	193	57	503
Clinical surveillance	19	11	29	19	11	29
**Total**	**362**	**75**	**1073**	**241**	**100**	**557**
Total (excl. drugs)	334	48	1038	213	74	522

Best estimates with 5th and 95th centiles, 2015 US$ millions*.

*See Supporting Information for the studies and regression models used to estimate economic and financial unit costs. The difference between economic and financial costs is explained in the methods.

Two to three years of clinical surveillance in the 12 known endemic countries adds about US$ 18 (11–29) million. The total economic cost benchmark is therefore US$ 362 (75–1073) million. Excluding drugs, the economic cost benchmark is US$ 334 (48–1038) million. The benchmark cost of clinical surveillance in the 71 countries requiring verification of the absence of the disease is about US$ 33 (7.5–59) million.

### Health effects

**Figure 3:**
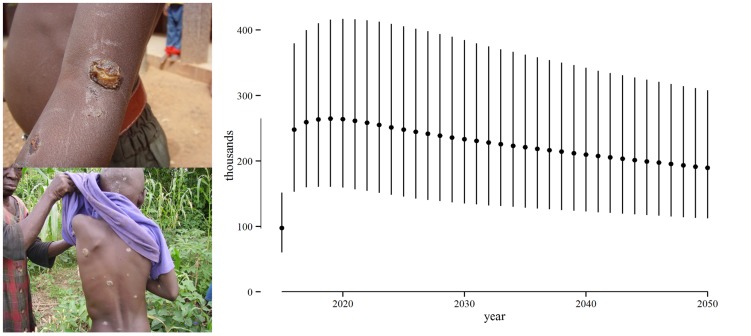
Years of life that could be lived without early-stage yaws, 2015–2050. Years of life lived without early-stage yaws due to the eradication campaign. Early-stage yaws includes primary and secondary stage cases, but excludes latent cases. Points represent best estimates (means) and the vertical lines represent the 90% credible interval. The line on the y-axis gives the range of best estimates over the period. Photo credit: Henri Asse.

**Figure 4:**
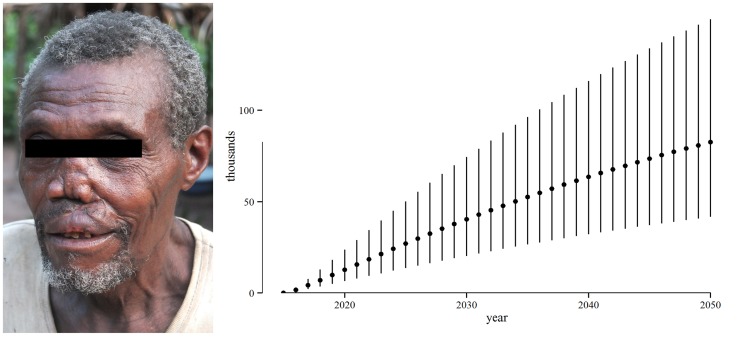
Years of life that could be lived without late-stage yaws, 2015–2050. Years of life lived without late-stage yaws due to the eradication campaign. Points represent best estimates (means) and the vertical lines represent the 90% credible interval. The line on the y-axis gives the range of best estimates over the period. Clinical symptoms of late-stage yaws are depicted on the left. Photo credit: MSF Epicentre.

In the absence of an eradication campaign in the 12 known endemic countries, the number of years of life lived with early-stage yaws would be 13 (7.5–21) million in the period 2015–2050. See Figure 3. The credible intervals are large, reflecting our conservative choice of parameter distributions. The number of years of life lived with late-stage yaws would be 3.0 (1.5–5.4) million. See Figure 4. This amounts to 16 (9.4–26) million years of life lived with yaws symptoms and 1.6 (0.8–2.9) million DALYs.

Given that tertiary yaws is irreversible, eradication would avert most but not all of this burden, leaving 1.0 (0.6–1.7) million years of life lived with yaws symptoms and 0.3 (0.1–0.5) million DALYs.

Due to the eradication campaign, 13 (7.3–20) million years of life would be lived without early-stage yaws and 2.3 (1.1–4.2) million years of life without late-stage yaws. 1.3 (0.6–2.4) million DALYs would be averted.

### Cost-effectiveness

**Figure 5:**
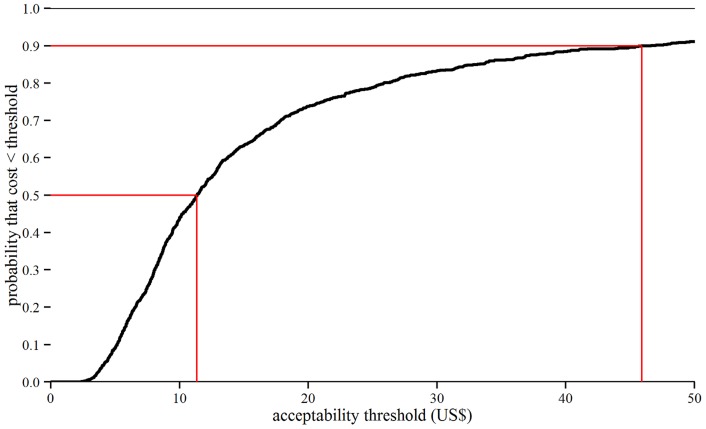
Cost per year of life that could be lived without yaws symptoms: Cost-effectiveness acceptability curve. Cost per year of life lived without yaws symptoms due to the eradication campaign. Symptoms include those of primary, secondary (excluding latent) or tertiary yaws. The x-axis gives a range of possible thresholds for the acceptability of the cost per year of life lived without yaws symptoms (in US$). The y-axis gives the probability that the model is consistent with yaws eradication being cost-effective at a given threshold.

The total economic cost per year of life lived without yaws symptoms is estimated at US$ 26 (4.2–78) for the 12 known endemic countries. There are no established thresholds for the acceptability of the cost per year of life lived without yaws symptoms, but Figure 5 shows that the probability of acceptability would exceed 50% at a threshold of US$ 11.35 and 90% at US$ 46.

The cost per DALY averted is US$ 324 (47–936). The interval is large, but well below WHO thresholds for cost-effectiveness of three times GDP per capita [28]. We estimate GCP per capita of US$ 733 (2005 US$) for the 74 million people living under conditions favorable to yaws infection, and GDP per capita of US$ 799 for the 12 known endemic countries as a whole. Even under the most conservative assumptions, yaws eradication is cost-effective.

### Affordability

Populations at risk for yaws are poor, but the areas in which they live are economically productive. G-Econ data suggest a GCP of US$ 37 400 per square kilometer (2005 US$) and US$ 51 billion in total, or 17% of GDP in the 12 known endemic countries. The best estimate of the cost of eradication represents less than 0.5% of GCP and less than 0.1% of GDP. It would appear to be affordable from the perspective of the economy as a whole.

Recall also the difference between economic and financial costs. In practice, many costs will be covered by existing Ministry of Health staff and assets such as vehicles. Excluding drugs and Ministry of Health staff and assets, the financial cost of yaws eradication could be as little as US$ 213 (74–522) million in the 12 endemic countries.

## Discussion

This paper provides the first economic evaluation of yaws eradication. It is largely prospective and, as a consequence, conclusions are limited by our uncertainty about many parameters, for both costs and effects. Most but not all of this uncertainty was reflected in a probabilistic sensitivity analysis. The number and distribution of people at risk for yaws needs to be better mapped, to better model health effects, certainly, but also costs. Uncertainty around populations at risk is not fully reflected in the regression model estimates of the unit cost of delivery. In the case of DRC, for example, we may have overestimated economies of scale and underestimated logistical difficulty. The cost benchmarks presented in this study are certainly not a substitute for country plans and budgets. Recall that we have not included the cost of molecular tests to confirm eradication, though this may only need to start once clinical cases are no longer being found.

There is also uncertainty about the 71 countries of unknown endemicity. While we have included the cost of clinical surveillance in these countries, we have not included the cost of serological and molecular tests, much less the cost of TCT, that might be incurred if clinical yaws cases are identified in any one of them. Given that some of these countries share borders with the 12 countries of known endemicity, cross-border issues will incur, at the very least, some coordination costs. That said, we have also not included the health benefits that would accrue in these 71 countries, so the influence that their inclusion would have on the cost-effectiveness result is ambiguous.

The cost of the “end game” of any eradication effort is uncertain, with the emergence of complexities requiring some local adaptation of global strategies [29]. Much of the uncertainty will be resolved as endemic countries begin or, in the case of the pilot sites, continue to implement the program. Nonetheless, there are good reasons to believe that a global yaws eradication campaign could be established with a relatively modest investment in the period 2015–2020 – about US$ 100–500 million in the 12 endemic countries. The real cost of waiting for more roads (the end of poverty) would be millions of years of life lived with disability and disfigurement due to yaws. Yaws eradication appears to be very cost-effective under reasonable assumptions about its cost and effects, and even under optimistic scenarios of poverty reduction.

The main question that remains is how to finance the next phase of implementation. The governments of endemic countries are encouraged to take ownership of national elimination efforts. But the global public good of yaws eradication will likely require global financing. The cost to the public sector would be significantly reduced by drug donations from pharmaceutical companies, similar to those being made for other preventable NTDs. Donations of diagnostic tests would also help. At least as important is the catalytic effect that these in-kind donations could have. Financial and in-kind resources could be better harnessed from the extractive industries (e.g. mining, logging) and others (e.g. cocoa and coffee). These are industries with operations on or near the resource-rich lands where resource-poor populations still live with yaws. There is already some precedent for mining company support to yaws eradication implementation and research in Lihir [30].

If endemic countries and their financing partners deliver within the range of costs and effects considered in this study, yaws eradication will be cost-effective relative to WHO thresholds. Of course, the case for investment in yaws eradication does not rest on cost-effectiveness alone. Policy-makers may be confronted with choices between public health interventions of similar cost-effectiveness relative to WHO thresholds. In the context of universal health coverage, priority-setting should consider also equity, with priority given to the worse off. There is no doubt that efforts to eradicate yaws will benefit some of the world's least well off citizens. Yaws eradication should be seen as complementary to universal health coverage and shared prosperity on the post-2015 development agenda.

## Supporting Information

Table S1 Studies of the cost of mass drug administration to control and eliminate other Neglected Tropical Diseases. 25 studies identified during a review of the literature on the cost of mass drug administration (MDA) to control and eliminate other NTDs: lymphatic filariasis (LF), schistosomiasis, soil-transmitted helminthiasis (STH), onchocerciasis and trachoma.
(DOCX)

Table S2 Regression models for the cost of mass drug administration (excluding drugs) per person treated. Regression models fitted using data from the 25 studies referenced in Table S1. Financial (F) and economic (E) costs include: planning, mapping and training activities (F&E), drug shipment (F&E), vehicles that were rented (F&E) or borrowed from other programs (E), fuel and vehicle maintenance (F&E), per diems (F&E), project staff salaries (F&E), Ministry of Health staff time (E), office space (E), utilities (F&E) and supplies (F&E). Both costs exclude drugs and volunteer time. 95% confidence intervals for the regression coefficients are in square brackets. *p<0.15, **p<0.10, ***p<0.05, ****p<0.01.
(DOCX)

Table S3 Benchmark costs for Total Community Treatment for yaws (excluding drugs) per person treated. Estimates of unit cost (2012 US$) obtained using the regression models reported in Table S2 and country-specific data on populations at risk of yaws, GDP per capita and population density. Financial (F) and economic (E) costs include: planning, mapping and training activities (F&E), drug shipment (F&E), vehicles that were rented (F&E) or borrowed from other programs (E), fuel and vehicle maintenance (F&E), per diems (F&E), project staff salaries (F&E), Ministry of Health staff time (E), office space (E), utilities (F&E) and supplies (F&E). Both costs exclude drugs and volunteer time. Best estimates are the mean, and low and high estimates are the 5^th^ and 95^th^ centile values, respectively.
(DOCX)
