# Supplementary material for: Where the Road Ends, Yaws Begins? The Cost-effectiveness of Eradication versus More Roads
Source: PLoS Negl Trop Dis. 2014 Sep 25;8(9):e3165. doi: 10.1371/journal.pntd.0003165 (PMC4177754; doi:10.1371/journal.pntd.0003165)
Supplement: Table S1 — Studies of the cost of mass drug administration to control and eliminate other Neglected Tropical Diseases. 25 studies identified during a review of the literature on the cost of mass drug administration (MDA) to control and eliminate other NTDs: lymphatic filariasis (LF), schistosomiasis, soil-transmitted helminthiasis (STH), onchocerciasis and trachoma. (DOCX) [file pntd.0003165.s001.docx]

**Supporting Information**

**Table S1. Studies of the cost of mass drug administration to control and eliminate other Neglected Tropical Diseases**

1. Amarillo M, Belizario VJ, Panelo C, Sison S, de Leon W, et al. (2009) Cost of mass drug administration for filariasis elimination in the province of Sorsogon, Philippines. Acta Med Philipp 43: 23–28.

2. Brooker S, Kabatereine NB, Fleming F, Devlin N (2008) Cost and cost-effectiveness of nationwide school-based helminth control in Uganda: intra-country variation and effects of scaling-up. Health Policy Plan 23: 24–35. doi:10.1093/heapol/czm041.

3. Curtale F, Abd-el Wahab Hassanein Y, El Wakeel A, Barduagni P, Savioli L (2003) The School Health Programme in Behera: an integrated helminth control programme at Governorate level in Egypt. Acta Trop 86: 295–307.

4. Evans D, McFarland D, Adamani W, Eigege A, Miri E, et al. (2011) Cost-effectiveness of triple drug administration (TDA) with praziquantel, ivermectin and albendazole for the prevention of neglected tropical diseases in Nigeria. Ann Trop Med Parasitol 105: 537–547. doi:10.1179/2047773211Y.0000000010.

5. Frick KD, Lietman TM, Holm SO, Jha HC, Chaudhary JS, et al. (2001) Cost-effectiveness of trachoma control measures: comparing targeted household treatment and mass treatment of children. Bull World Health Organ 79: 201–207.

6. Gabrielli A-F, Touré S, Sellin B, Sellin E, Ky C, et al. (2006) A combined school- and community-based campaign targeting all school-age children of Burkina Faso against schistosomiasis and soil-transmitted helminthiasis: performance, financial costs and implications for sustainability. Acta Trop 99: 234–242. doi:10.1016/j.actatropica.2006.08.008.

7. Goldman AS, Brady MA, Direny A, Desir L, Oscard R, et al. (2011) Costs of integrated mass drug administration for neglected tropical diseases in Haiti. Am J Trop Med Hyg 85: 826–833. doi:10.4269/ajtmh.2011.10-0635.

8. Goldman AS, Guisinger VH, Aikins M, Amarillo MLE, Belizario VY, et al. (2007) National mass drug administration costs for lymphatic filariasis elimination. PLoS Negl Trop Dis 1: e67. doi:10.1371/journal.pntd.0000067.

9. Guyatt H (2003) The cost of delivering and sustaining a control programme for schistosomiasis and soil-transmitted helminthiasis. Acta Trop 86: 267–274.

10. Hodges MH, Smith SJ, Fussum D, Koroma JB, Conteh A, et al. (2010) High coverage of mass drug administration for lymphatic filariasis in rural and non-rural settings in the Western Area, Sierra Leone. Parasit Vectors 3: 120. doi:10.1186/1756-3305-3-120.

11. Kabatereine NB, Tukahebwa EM, Kazibwe F, Twa-Twa JM, Barenzi JFZ, et al. (2005) Soil-transmitted helminthiasis in Uganda: epidemiology and cost of control. Trop Med Int Health 10: 1187–1189. doi:10.1111/j.1365-3156.2005.01509.x.

12. Kolaczinski JH, Robinson E, Finn TP (2011) The cost of antibiotic mass drug administration for trachoma control in a remote area of South Sudan. PLoS Negl Trop Dis 5: e1362. doi:10.1371/journal.pntd.0001362.

13. Krishnamoorthy K, Ramu K, Srividya A, Appavoo NC, Saxena NB, et al. (2000) Cost of mass annual single dose diethylcarbamazine distribution for the large scale control of lymphatic filariasis. Indian J Med Res 111: 81–89.

14. Leslie J, Garba A, Boubacar K, Yayé Y, Sebongou H, et al. (2013) Neglected tropical diseases: comparison of the costs of integrated and vertical preventive chemotherapy treatment in Niger. Int Health 5: 78–84. doi:10.1093/inthealth/ihs010.

15. Leslie J, Garba A, Oliva EB, Barkire A, Tinni AA, et al. (2011) Schistosomiasis and soil-transmitted helminth control in Niger: cost effectiveness of school based and community distributed mass drug administration [corrected]. PLoS Negl Trop Dis 5: e1326. doi:10.1371/journal.pntd.0001326.

16. McFarland D, Menzies N, Njoumemi Z, Onwujekwe O (2005) Study of cost per treatment with ivermectin using the CDTI strategy. African Programme for Onchocerciasis Control (APOC).

17. McLaughlin SI, Radday J, Michel MC, Addiss DG, Beach MJ, et al. (2003) Frequency, severity, and costs of adverse reactions following mass treatment for lymphatic filariasis using diethylcarbamazine and albendazole in Leogane, Haiti, 2000. Am J Trop Med Hyg 68: 568–573.

18. Montresor A, Zin TT, Padmasiri E, Allen H, Savioli L (2004) Soil-transmitted helminthiasis in Myanmar and approximate costs for countrywide control. Trop Med Int Health 9: 1012–1015. doi:10.1111/j.1365-3156.2004.01297.x.

19. Onwujekwe O, Chima R, Shu E, Okonkwo P (2002) Community-directed treatment with ivermectin in two Nigerian communities: an analysis of first year start-up processes, costs and consequences. Health Policy 62: 31–51.

20. Oshish A, AlKohlani A, Hamed A, Kamel N, AlSoofi A, et al. (2011) Towards nationwide control of schistosomiasis in Yemen: a pilot project to expand treatment to the whole community. Trans R Soc Trop Med Hyg 105: 617–627. doi:10.1016/j.trstmh.2011.07.013.

21. Phommasack B, Saklokham K, Chanthavisouk C, Nakhonesid-Fish V, Strandgaard H, et al. (2008) Coverage and costs of a school deworming programme in 2007 targeting all primary schools in Lao PDR. Trans R Soc Trop Med Hyg 102: 1201–1206. doi:10.1016/j.trstmh.2008.04.036.

22. Ramzy RMR, Goldman AS, Kamal HA (2005) Defining the cost of the Egyptian lymphatic filariasis elimination programme. Filaria J 4: 7. doi:10.1186/1475-2883-4-7.

23. Sinuon M, Tsuyuoka R, Socheat D, Montresor A, Palmer K (2005) Financial costs of deworming children in all primary schools in Cambodia. Trans R Soc Trop Med Hyg 99: 664–668. doi:10.1016/j.trstmh.2004.12.004.

24. Talaat M, Evans DB (2000) The costs and coverage of a strategy to control schistosomiasis morbidity in non-enrolled school-age children in Egypt. Trans R Soc Trop Med Hyg 94: 449–454.

25. Turner HC, Osei-Atweneboana MY, Walker M, Tettevi EJ, Churcher TS, et al. (2013) The cost of annual versus biannual community-directed treatment of onchocerciasis with ivermectin: Ghana as a case study. PLoS Negl Trop Dis 7: e2452. doi:10.1371/journal.pntd.0002452.
