# Supplementary material for: Where the Road Ends, Yaws Begins? The Cost-effectiveness of Eradication versus More Roads
Source: PLoS Negl Trop Dis. 2014 Sep 25;8(9):e3165. doi: 10.1371/journal.pntd.0003165 (PMC4177754; doi:10.1371/journal.pntd.0003165)
Supplement: Table S2 — Regression models for the cost of mass drug administration (excluding drugs) per person treated. Regression models fitted using data from the 25 studies referenced in Table S1. Financial (F) and economic (E) costs include: planning, mapping and training activities (F&E), drug shipment (F&E), vehicles that were rented (F&E) or borrowed from other programs (E), fuel and vehicle maintenance (F&E), per diems (F&E), project staff salaries (F&E), Ministry of Health staff time (E), office space (E), utilities (F&E) and supplies (F&E). Both costs exclude drugs and volunteer time. 95% confidence intervals for the regression coefficients are in square brackets. *p<0.15, **p<0.10, ***p<0.05, ****p<0.01. (DOCX) [file pntd.0003165.s002.docx]

**Supporting Information**

**Table S2. Regression models for the cost of mass drug administration (excluding drugs) per person treated**

|  | (1) | (2) |
| --- | --- | --- |
|  | (log) Economic unit cost, excluding volunteer time, 2012 US$ | (log) Financial unit cost, 2012 US$ |
| (log) Population treated | -0.514^****^ [-0.68,-0.35] | -0.785^****^ [-0.97,-0.60] |
|  |  |  |
| (log) GDP per capita, 2012 US$ | 0.0405 [-1.26,1.34] | 0.654^**^ [-0.09,1.40] |
|  |  |  |
| (log) Population density (per km2) | -1.809^*^ [-4.31,0.69] | -4.546^****^ [-6.54,-2.56] |
|  |  |  |
| Constant | 14.36^***^ [1.54,27.19] | 24.69^****^ [14.58,34.79] |
| Observations | 103 | 97 |
| Groups | 57 | 67 |
| *R*^2^ | 0.578 | 0.862 |

Regression models fitted using data obtained from The 25 identified studies are referenced in Table S1. Financial (F) and economic (E) costs include: planning, mapping and training activities (F&E), drug shipment (F&E), vehicles that were rented (F&E) or borrowed from other programs (E), fuel and vehicle maintenance (F&E), per diems (F&E), project staff salaries (F&E), Ministry of Health staff time (E), office space (E), utilities (F&E) and supplies (F&E). Both costs exclude drugs and volunteer time. 95% confidence intervals for the regression coefficients are in square brackets. * p < 0.15, ** p < 0.10, *** p < 0.05, **** p < 0.01
