# Supplementary material for: Where the Road Ends, Yaws Begins? The Cost-effectiveness of Eradication versus More Roads
Source: PLoS Negl Trop Dis. 2014 Sep 25;8(9):e3165. doi: 10.1371/journal.pntd.0003165 (PMC4177754; doi:10.1371/journal.pntd.0003165)
Supplement: Table S3 — Benchmark costs for Total Community Treatment for yaws (excluding drugs) per person treated. Estimates of unit cost (2012 US$) obtained using the regression models reported in Table S2 and country-specific data on populations at risk of yaws, GDP per capita and population density. Financial (F) and economic (E) costs include: planning, mapping and training activities (F&E), drug shipment (F&E), vehicles that were rented (F&E) or borrowed from other programs (E), fuel and vehicle maintenance (F&E), per diems (F&E), project staff salaries (F&E), Ministry of Health staff time (E), office space (E), utilities (F&E) and supplies (F&E). Both costs exclude drugs and volunteer time. Best estimates are the mean, and low and high estimates are the 5th and 95th centile values, respectively. (DOCX) [file pntd.0003165.s003.docx]

**Supporting Information**

**Table S3. Benchmark costs for Total Community Treatment for yaws (excluding drugs) per person treated**

|  | **Economic** | | | **Financial** | | |
| --- | --- | --- | --- | --- | --- | --- |
| **Country** | **Best** | **Low** | **High** | **Best** | **Low** | **High** |
| Benin | 3.69 | 0.21 | 14.02 | 2.39 | 0.71 | 5.70 |
| Cameroon | 1.25 | 0.14 | 3.67 | 0.69 | 0.28 | 1.35 |
| Central African Republic | 2.70 | 0.22 | 9.67 | 1.36 | 0.37 | 3.13 |
| Congo | 10.41 | 0.10 | 38.65 | 5.51 | 0.86 | 15.82 |
| Cote d'Ivoire | 0.20 | 0.06 | 0.45 | 0.05 | 0.03 | 0.10 |
| Democratic Republic of the Congo | 0.21 | 0.04 | 0.54 | 0.02 | 0.01 | 0.03 |
| Ghana | 0.22 | 0.05 | 0.60 | 0.07 | 0.03 | 0.14 |
| Indonesia | 8.06 | 0.08 | 32.47 | 5.68 | 1.01 | 15.64 |
| Papua New Guinea | 6.22 | 0.09 | 31.54 | 5.19 | 0.93 | 15.78 |
| Solomon Islands | 4.76 | 0.13 | 19.83 | 3.44 | 0.83 | 8.67 |
| Togo | 3.10 | 0.23 | 11.41 | 1.88 | 0.55 | 4.64 |
| Vanuatu | 9.01 | 0.11 | 33.20 | 6.34 | 1.09 | 17.66 |

Estimates of unit cost (2012 US$) obtained using the regression models reported in Table S2 and country-specific data on populations at risk of yaws, GDP per capita and population density. Financial (F) and economic (E) costs include: planning, mapping and training activities (F&E), drug shipment (F&E), vehicles that were rented (F&E) or borrowed from other programs (E), fuel and vehicle maintenance (F&E), per diems (F&E), project staff salaries (F&E), Ministry of Health staff time (E), office space (E), utilities (F&E) and supplies (F&E). Both costs exclude drugs and volunteer time. Best estimates are the mean, and low and high estimates are the 5^th^ and 95^th^ centile values, respectively.
